# Near-optimal integration of facial form and motion

**DOI:** 10.1038/s41598-017-10885-y

**Published:** 2017-09-08

**Authors:** Katharina Dobs, Wei Ji Ma, Leila Reddy

**Affiliations:** 1 Université de Toulouse, Centre de Recherche Cerveau et Cognition, Université Paul Sabatier, Toulouse, France; 2 CNRS, UMR 5549, Faculté de Médecine de Purpan, Toulouse, France; 3 New York University, Center for Neural Science and Department of Psychology, New York, New York, USA

## Abstract

Human perception consists of the continuous integration of sensory cues pertaining to the same object. While it has been fairly well shown that humans use an optimal strategy when integrating low-level cues proportional to their relative reliability, the integration processes underlying high-level perception are much less understood. Here we investigate cue integration in a complex high-level perceptual system, the human face processing system. We tested cue integration of facial form and motion in an identity categorization task and found that an optimal model could successfully predict subjects’ identity choices. Our results suggest that optimal cue integration may be implemented across different levels of the visual processing hierarchy.

## Introduction

In complex and dynamic environments, the integration of multiple sensory cues arising from the same object is essential for accurate perception. The optimal strategy is to weight these cues in proportion to their reliability^1, 2^. Humans employ this strategy when combining multiple low-level cues within^3–5^ and across^6–8^ modalities, but less is known about mechanisms for integration in high-level perception. For example, faces convey identity information through static (e.g., facial form) and dynamic cues^9, 10^. Coherent perception of facial identity would benefit from integrating such cues^11–14^.

Here, we asked how the human visual system integrates information provided by facial form and motion to categorize faces. Specifically, subjects categorized animated avatar faces, that could be independently manipulated in facial form and motion, into two previously learned identities based on facial form, motion and both cues combined. Similar to studies based on low-level stimuli, we expected that subjects integrate facial form and motion cues in an optimal fashion. One of the predictions of the optimal cue integration model is that subjects, on a trial-to-trial basis, reweigh a cue when its reliability changes. To test this prediction, we introduced an additional manipulation in which the facial form was made “old” thereby reducing its reliability. Finally, we compared three models that differ in how the visual system integrates facial form and motion information: (1) optimally, (2) by using only the most reliable cue, or (3) by computing a simple average of both cues. We found that the optimal model predicted subjects’ identity choices best, suggesting that this principle governs both low- and high-level perception.

## Results

### Behavioural cue integration

**Figure 1:**
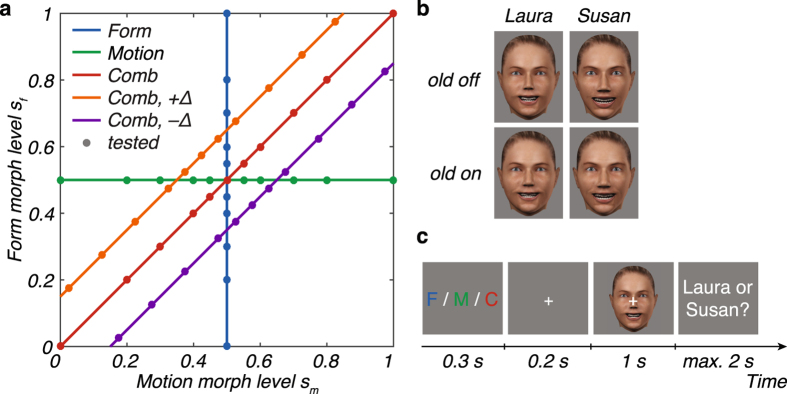
Experimental stimuli and task. (**a**) Experimental conditions. For each condition, stimuli were sampled from 11 form and motion morph levels *s*_f_ and *s*_m_, respectively. In the form condition, the form morph level varied while the motion morph level was kept constant at 0.5 (“Form”; blue line), and vice versa for the motion condition (“Motion”; green line). In combined-cue conditions, motion and form morph levels were either morphed by the same amount (“Comb”; red line) or differed slightly (“Comb, +Δ”: Form > Motion; orange line; “Comb, −Δ”: Form < Motion; purple line). Markers represent the sampled morph levels tested in the experiment. (**b**) Representative frames of the two “basic” (corresponding to the stimuli at (0,0) and (1,1) in panel a) dynamic face stimuli “Laura” and “Susan” (“old off”; upper row) and their “old” morphs (“old on”; lower row). Dynamic face stimuli were designed in Poser 2012 (SmithMicro, Inc., Watsonville, CA, USA). (**c**) Trial sequence. Subjects performed an identity categorization task (i.e., 2AFC) based on form (letter ‘F’), motion (letter ‘M’) or both combined (letter ‘C’) as indicated by a cue at the beginning of a trial. On each trial, one of the 1 s stimuli, sampled from the stimulus space (panel a) was presented followed by an inter-trial interval (ITI) of max. 2 s.

We probed cue integration behaviour in an identity categorization task. We used two facial identities, “Laura” and “Susan”, with distinct facial form and motion, as dynamic face stimuli (Fig. 1b, upper row “old off”). After an initial familiarization phase, followed by a discrimination test of these two identities (for details, see Methods and Supplementary Information), subjects (*n* = 22) performed an identity categorization task. Similar to studies on low-level integration, we tested cue integration based on form, motion or combined cues in separate blocks. On each trial, subjects categorized a dynamic face stimulus (1 s) as Laura or Susan. We used 11 morph levels, sampled from a continuum between Laura and Susan (represented by 0 and 1, respectively; Fig. 1a). In form blocks, dynamic face stimuli consisted of form morphs presented with the average facial motion, and vice versa for motion blocks (Fig. 1a, “Form” and “Motion”). In combined blocks, both cues were either morphed by the same amount (combined congruent; Fig. 1a, “Comb”) or differed slightly such that the stimulus contained more form than motion (Δ = +0.15) or more motion than form (Δ = −0.15) information about Susan (combined incongruent; Fig. 1a, “Comb, +Δ”, “Comb, −Δ”, respectively). Importantly, during debriefing, none of the subjects reported that they noticed a conflict between cues. We refer to these conditions (i.e., single-cue and combined-cue conditions) as “old off” (see below for the “old on” conditions).

**Figure 2:**
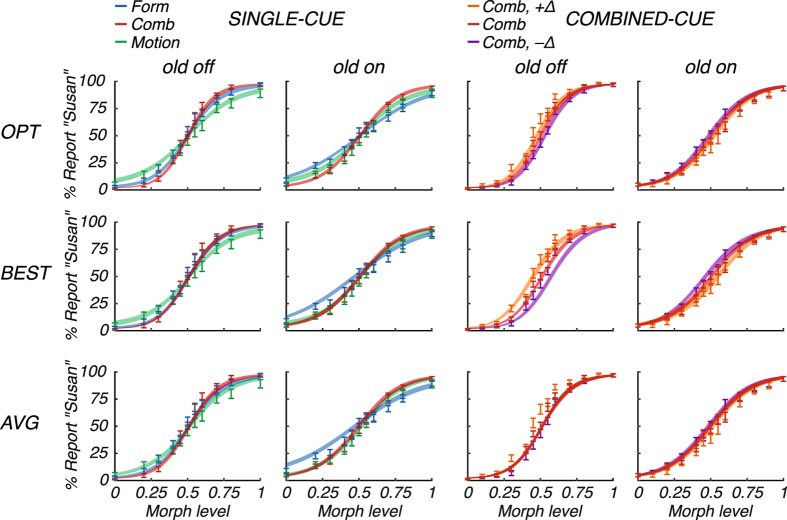
Psychometric curves and model fits. Mean percentage of “Susan” reports are shown for single cues (“Form” in blue, “Motion” in green; note that the combined-cue condition “Comb” is also shown for comparison) and for combined cues (“Comb” in red, “Comb, +Δ” in orange, and “Comb, −Δ” in purple), each separated for “old off” (first column) and “old on” (second column). Error bars and shaded areas represent ±1 s.e.m. across subjects (*n* = 22), for data and model fit, respectively. Fits are shown for the optimal model (OPT; upper row), the best-cue model (BEST; middle row) and the simple-average model (AVG; lower row).

One of the predictions of the optimal cue integration model is that on trials when the reliability of one cue is higher, subjects place a greater weight on that cue. To test this prediction, we introduced a manipulation in which each sampled stimulus in each condition was morphed into an average “old” face. We refer to these conditions as “old on”. The amount by which each stimulus was morphed into the average “old” face was set to 0.35, ensuring that the “old off” faces were highly distinct from the “old on” ones (see Methods). As described below, these “old on” conditions effectively reduced the reliability of the form information, but did not affect the motion information (see Methods). The percentage of “Susan” reports (Fig. 2) clearly increased with the amount of morph level, suggesting that subjects could discriminate the identities based on facial form, facial motion, and both cues combined, in “old off” and “old on” conditions. Visual inspection of the mean percentage of “Susan” reports in the single-cue conditions (Fig. 2, left columns) suggests that form is less reliable in the “old off” than the “old on” condition, in line with a smaller estimated standard deviation for form in the “old off” than in the “old on” conditions (*σ*_f_: 0.18, [0.14, 0.31] (median and IQR across subjects); *σ*_f,old_: 0.21, [0.19, 0.27]; see Supplementary Information). Accordingly, the psychometric curves for “old off” and “old on” seem shifted with respect to each other in the combined incongruent conditions (Fig. 2, right columns): in the “old off” condition, subjects tended to report “Susan” at lower morph levels when there is more form than motion information (Comb, +Δ; orange), as can be seen by a shift to the left compared to the congruent condition (Comb; red), whereas the opposite can be seen for “old on”. These results indeed suggest that subjects, on a trial-to-trial basis, place a greater weight on form in the “old off” conditions, while they rely more on motion in the “old on” conditions, as predicted by the optimal model.

### Optimal model fit

We next examined whether subjects’ identity choices followed the optimal model. We assume that each stimulus gives rise to a noisy internal measurement, described by a Gaussian distribution with standard deviation *σ*_m_ for motion and *σ*_f_ for form. Many cue integration studies use the standard deviations estimated from the single-cue conditions to predict behaviour in the combined-cue conditions. This approach, which necessitates a large number of trials, treats the single-cue and combined-cue conditions differently, using only the latter to evaluate goodness of fit. Due to the relatively low number of trials in this experiment, we instead fit all conditions (single-cue and combined-cue) jointly using maximum-likelihood estimation; results of a predictive analysis are reported below.

In particular, we fitted five parameters (the standard deviations for facial motion *σ*_m_, facial form for “old off” *σ*_f_ and “old on” *σ*_f,old_, the category boundary *b*, and a lapse rate *λ*) to each subject’s individual-trial responses. The median maximum-likelihood estimates of the parameters were 0.17 (IQR: [0.14, 0.22]) for *σ*_f_, 0.24 (IQR: [0.19, 0.30]) for *σ*_f,old_, 0.28 (IQR: [0.21, 0.35]) for *σ*_m_, 0.51 (IQR: [0.46, 0.55]) for *b*, and 0.04 (IQR: [0.01, 0.05]) for *λ*. Figure 2 shows the fit of the optimal model to the psychometric curves.

### Model comparison

**Figure 3:**
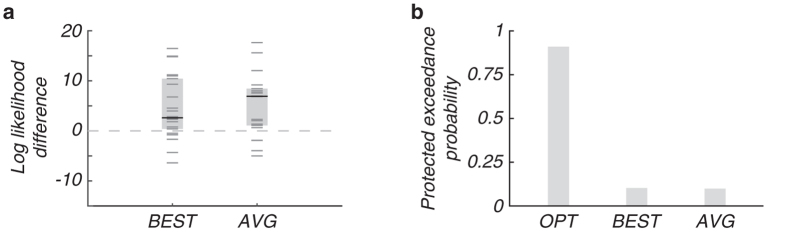
Model comparison. (**a**) Median differences (black lines) in log likelihood between the optimal and the best-cue (BEST) model, and the optimal and the simple-average (AVG) model. Boxes show IQR and grey lines represent single subject’s raw data (*n* = 22). (**b**) Protected exceedance probabilities of the models.

We compared the optimal model to two models that differed in the way facial form and motion information are integrated: in the best-cue model, the observer uses only the most reliable cue (Fig. 2, BEST, middle row), and in the simple-average model, the observer forms a simple average based on both cues (i.e., neglecting each cue’s reliability; Fig. 2, AVG, lower row). For each model, we fitted data from all conditions jointly using maximum-likelihood estimation. While each of the three models fitted the single-cue data similarly well (Fig. 2, left columns), clear differences were found in the combined-cue conditions (Fig. 2, right columns). Comparing the models’ maximum log likelihoods revealed that the optimal model is more likely than the best-cue model with a median difference of 2.50 (IQR: [0.28, 10.33]; *z* = 2.68, *p* = 0.007; two-sided Wilcoxon signed-rank test; Fig. 3a, BEST) and is more likely than the simple-average model (6.80, IQR: [0.98, 8.32]; *z* = 3.13, *p* = 0.002; Fig. 3a, AVG). All models had the same number of free parameters, so no correction for the number of free parameters is needed.

While the optimal model overall outperforms the alternative models and provides a good fit to the psychometric curves, some small deviations from the data can be observed. These could be due to different subjects following different models. A question of interest then is what the prevalence of each of the models in the population might be. To examine this, we applied a novel random-effects method for Bayesian model selection^15^ that returns the “protected exceedance probability” *p*_pxp_ of each model, which is the probability that a given model is the most frequent model in the population above and beyond chance (Fig. 3b). The optimal model had a much higher exceedance probability (*p*_pxp_ = 0.819; OPT) than the best-cue (*p*_pxp_ = 0.092; BEST) and simple-average models (*p*_pxp_ = 0.088; AVG). This method also returns the posterior probabilities of the three models on an individual-subject basis. The posterior probability was highest for the optimal model in 15 out of 22 subjects, highest for the best-cue model in three, and highest for the simple-average model in four subjects.

### Predictive analysis

Despite our relatively low number of trials, we still tested how well the parameters estimated from the single-cue conditions could predict (without any additional parameter fitting) the subject’s choices in the combined-cue condition. The overall log likelihood was still the sum of the log likelihoods in the single-cue and the combined-cue conditions. Similar to the joint fitting, the optimal model was more likely than the best-cue model (1.82, [−3.55, 8.62] (median difference, IQR across subjects)), but the difference was not significant (*z* = 0.86, *p* > 0.250, two-sided Wilcoxon signed-rank test). By contrast, the optimal model did not differ from the simple-average model (−0.73, [−11.08, 5.85]; *z* = −0.67, *p* > 0.250). Comparison between this predictive analysis and the joint fitting procedure reveals overall higher log likelihoods of the latter (optimal: 23.31, [6.23, 43.07], *z* = 4.11, *p* < 0.001; best-cue: 18.67, [11.59, 44.80]; *z* = 4.11, *p* < 0.001; simple-average: 17.03, [4.43, 35.51], *z* = 4.11, *p* < 0.001); we therefore consider the results of the joint fitting procedure to be our main results.

### Optimal model with incorrect beliefs

The optimal model assumes that observers have complete knowledge of the task structure, except for the fact that they are not aware of the presence of “incongruent” trials. While this model is ideal, it is possible that subjects do not realize that the “old” face is a morph between Laura and Susan. That is, they are not aware that we have morphed into the perceptual “old” average of Laura and Susan to create the “old” faces. We thus tested a variant of the optimal model for which observers assume that the “old” morphs are pure “old” versions of the facial identities (e.g., the “old” version of Laura is pure Laura and does not contain any information about Susan). This “optimal model with incorrect beliefs” did not significantly differ from the optimal model (median log likelihood difference: 0.59, IQR: [−0.34, 2.23]; *z* = 1.64, *p* = 0.101, two-sided Wilcoxon signed-rank test). As the optimal model, it outperformed the best-cue model (2.31, IQR: [0.02, 7.02]; *z* = 2.78, *p* = 0.005; two-sided Wilcoxon signed-rank test) and the simple-average model (3.88, IQR: [1.00, 8.46]; *z* = 3.04, *p* = 0.002). Exact parameters and model fits are reported in the Supplemental Information.

## Discussion

In this study, we measured human observers’ identity categorization performance using well-controlled, computer-generated, dynamic face stimuli that systematically varied in the amount of identity information conveyed by facial form, facial motion, or both. We manipulated form reliability by morphing faces into an average “old” face. Subjects’ identity choices in this high-level task could arise from optimally integrating both cues on a trial-to-trial basis (optimal model), from using only the most reliable cue (best-cue model), or from performing a simple average of both cues (simple-average model). The optimal model accounted best for subjects’ choice behaviour in terms of goodness of fit (log likelihood) and protected model exceedance probability. Our results extend cue integration studies on low-level perception by showing that a high-level cognitive process, such as the recognition of a face, can be successfully predicted by Bayesian inference. Moreover, our results strengthen previous studies that showed qualitatively that form and motion information are combined during object and face perception^14, 16^; and that audio and visual cues are integrated during multisensory face perception^17, 18^.

A major challenge in investigating cue integration during high-level perception is to create well-controlled stimuli that can be systematically manipulated in the amount of information conveyed by different cues. Here, we used a state-of-the-art motion retargeting and facial animation system to create well-controlled dynamic face stimuli^19^. The use of dynamic face stimuli to investigate cue integration has advantages over low-level stimuli. First, low-level stimuli such as blobs^6^ and dot clouds^20^ often lack ecological validity and necessitate the use of somewhat contrived backstories to induce subjects to integrate the cues. By contrast, dynamic faces represent a highly relevant ecological stimulus that humans encounter everyday. Second, an important assumption of the optimal model is that the cues that are to be integrated arise from a common source. An advantage of using faces as stimuli is that faces naturally contain several cues that clearly arise from the same high-level object. However, we cannot exclude the possibility that, despite our precautions and subjects’ reports, subjects’ visual systems still noticed something “odd” within the combined incongruent conditions; in this case, their behaviour might be better described by a causal inference model^21, 22^. Third, the use of facial form and facial motion as cues to identity is particularly interesting as these cues differ in their spatio-temporal dynamics. While form information is available immediately at the beginning of the stimulus, facial motion unfolds in time. In line with this difference in sensory evidence over time, we find differences in reaction times between the two cues (see Supplemental Information). Recent evidence has shown that under such conditions behaviour can be sub-optimal when reaction time is not taken into account^23, 24^. Here, we have not included reaction times in our optimal model. We thus need to interpret our results with caution, as they might change with a more complete model. Further work should investigate if subjects accumulate evidence optimally over time and across facial cues, and thereby better characterize the spatio-temporal dynamics of high-level cue integration behaviour.

The large number of experimental conditions needed to systematically investigate the integration of facial form and motion limited the number of identities we could use. Ideally, many different facial identities should be tested to rule out exemplar-specific strategies. However, a previous study based on very different face stimuli reported similar shifts in psychometric functions towards motion information when form reliability was reduced^14^, suggesting that the effects reported here indeed generalize to a wider range of stimuli. Our study goes beyond these previous findings by manipulating not only the form but also the motion cue, thus allowing us to quantitatively investigate the underlying integration mechanisms.

We used a linear morphing technique to generate our face stimuli and it is conceivable that this technique might not directly map onto a linear percept. While we have not validated the linear morphing technique, we note that the parametric motion and form space indeed monotonically increased with observers’ choice behaviour. Moreover, we found the bias *b*, estimated across all conditions, to match almost exactly the intermediate morph level (i.e., median morph level of 0.51).

To accomplish trial-by-trial weighing of cues, the optimal model assumes that the brain does not only represent stimulus estimates, but also the associated levels of uncertainty. This could for example be achieved through a probabilistic population code^25^, in which the population pattern of activity on each trial represents a probability distribution over the stimulus (here, morph level). Roughly speaking, the total activity in the population would – on a trial-to-trial basis – be inversely related to the observer’s uncertainty about the stimulus. Downstream “integrative” populations can then perform optimal cue integration by summing the single-cue population activities. With some modifications, this theory is in line with recent neurophysiological studies in monkeys^26, 27^.

With regard to dynamic face perception, our results have some implications for its underlying neural processing. Traditionally, two anatomically segregated pathways of processing for facial motion and facial form information have been proposed^12, 28–30^: a ventral pathway, including the occipital face area and the fusiform face area, processing facial form, and a dorsal pathway, including motion-processing areas in the medial temporal lobe and a posterior part of superior temporal sulcus (STS), processing facial motion. By contrast, and in line with more recent reports^11, 31–33^, our results suggest that facial form and facial motion information are integrated during the processing of facial identity. Several studies have suggested that STS may integrate facial form and motion information in humans^34–37^, and our results open the door to more quantitative investigations of the nature of this integrative process; in particular, it would be interesting to decode both estimates and uncertainty levels from multi-voxel activity in the STS^38^.

Overall, the human dynamic face processing system provides an excellent opportunity to understand cue integration in a complex, high-level perceptual system. Our results provide evidence that subjects’ choices in a facial cue integration task are near-optimal, suggesting flexibility and dynamic reweighting of facial cue representations. Moreover, our findings suggest that Bayesian inference is implemented at different levels of the visual processing hierarchy.

## Methods

### Participants

Twenty-two observers (15 female; age range 19–47 years) volunteered as subjects. Based on previous work^14^, we aimed to collect data from 20 subjects. We allowed 22 people to sign up in case of dropouts and stopped data collection after testing all those who did show up. All subjects had normal or corrected-to-normal vision and provided informed written consent prior to the experiment. The study was approved by the Comité d’Evaluation Ethique de l’INSERM in Paris, France and conducted in accordance with relevant guidelines and regulations.

### Stimuli and display

To create dynamic face stimuli that could be independently manipulated in facial form and facial motion, we used a motion-retargeting and facial animation procedure. Briefly, the procedure used to make these animations was as follows (for details see ref. 19). First, a “happy” facial expression was motion-recorded from two female actors following a previously validated and published procedure^19^. The two basic facial expressions started from a neutral expression that proceeded to the peak expression. Second, two basic avatar faces, referred to as “Laura” and “Susan” (Fig. 1b), were designed in Poser 2012 (SmithMicro, Inc., Watsonville, CA, USA) differing largely in size, shape and configuration of their facial features. To avoid introducing an identity cue in addition to form, the facial texture (e.g., skin colour, eye colour) was kept constant. Intermediate stimuli were created by linearly morphing from Laura to Susan. This was done either separately for facial form (keeping facial motion constant) and facial motion (keeping facial form constant), or for both cues combined (Fig. 1a). For the combined-cue conditions, the morph level of facial form and motion was either the same (i.e., combined congruent condition; Fig. 1a, red diagonal line) or differed by a small amount (i.e., combined incongruent conditions; Fig. 1a, orange and purple lines).

To test the effect of cue reliability, we reduced form reliability as follows: the two basic facial avatars and their intermediate form morphs were additionally morphed into an average “old” face by 35% (Fig. 1b). This “old” face was created by averaging Laura and Susan’s facial forms with weights 0.4 and 0.6, respectively, and applying an “old” morpher (morph level 0.35) provided in Poser to this average face. The value of 0.35 was chosen based on preliminary testing during the familiarization phase so that subjects clearly perceived the faces as “old” but were still able to discriminate Laura from Susan. Moreover, the weights of 0.4 and 0.6 were chosen so that the “old” face was perceived as an average.

Finally, the two basic avatar identities, the intermediate morphs, and the “old” morphs were animated by the motion-captured facial expressions and their intermediate motion morphs (for details about the motion retargeting procedure see ref. 39). Each animation was rendered as a Quicktime movie of 1 s duration (450 × 600 pixels, 30 frames at 60 Hz) in 3ds Max 2012 (Autodesk, Inc., San Rafael, CA, USA). All stimuli are freely available at https://osf.io/f7snh.

Stimuli were presented and responses recorded using PsychToolbox 3 for Matlab (http://www.psychtoolbox.org)^40, 41^. Observers were seated approximately 60 cm from a HP ZR2440w monitor (24 inch screen diagonal size, 1920 × 1200 pixel resolution; 60 Hz refresh rate). Face stimuli were scaled to a size of 9° × 12°.

### Procedure

The experimental procedure consisted of three phases: familiarization, training, and testing (for details see Supplementary Information). In the familiarization phase, subjects were familiarized with two facial identities “Laura” and “Susan” and their basic facial forms and facial motions (Fig. 1b, upper row “old off”) while performing a same-different task on each identity separated in two blocks for about 10 min. On each trial, a sequence of two dynamic face stimuli (1 s each) was presented: the “basic” identity (e.g., 100% Laura’s facial form and motion) followed by the same (“same” trials) or a slightly changed (“different” trials) stimulus. “Different” trials consisted of the same basic identity but morphed into an average “old” face (Fig. 1b, lower row “old on”), and the amount of morph level (0.05 morph steps from 0.05 to 1) was controlled by a Quest staircase procedure. Subjects received feedback at the end of each trial.

In the training phase, we probed learning of the two identities in an identity discrimination task for about 10 min. Subjects performed a two-alternative forced-choice task (2AFC; i.e., “Laura or Susan?”) based on facial form (i.e., facial motion was rendered uninformative by showing the average facial motion), facial motion (i.e., facial form was uninformative by showing the average facial form) or both cues combined in three separated blocks. On each trial, one basic (i.e., 100% form with average motion; 100% motion with average form; or 100% form and motion) dynamic face stimulus (1 s) was presented. Feedback was given. Note that neither intermediate stimuli nor “old” faces were shown during training.

Finally, we probed cue integration behaviour in an identity categorization task for about 70 min. Similar to studies on low-level integration, we tested cue integration based on form, motion or combined cues in separate blocks. On each trial, subjects performed a 2AFC reaction time task (i.e., “Laura or Susan?”) in which they could respond during or up to 2 s after the presentation of one dynamic face stimulus (stimulus duration 1 s). For an analysis of the reaction times see Supplementary Information. No feedback was provided. For each condition, 11 morph levels were sampled from a morph continuum between Laura and Susan (i.e., representing 0 and 1, respectively; Fig. 1a). In form blocks, dynamic face stimuli consisted of form morphs combined with the average facial motion, and vice versa for motion blocks (Fig. 1a, “Form” and “Motion”). In combined blocks, both cues were either morphed by the same amount (Fig. 1a, “Comb”) or differed slightly such that the stimulus contained more form than motion (Δ = +0.15) or more motion than form (Δ = −0.15) information about Susan (Fig. 1a, “Comb, +Δ”, “Comb, −Δ”, respectively). Importantly, in debriefing after the experiment, none of the subjects reported that they noticed a conflict between cues. We refer to these conditions (i.e., single-cue and combined-cue conditions) as “old off”.

We additionally introduced a manipulation in which each sampled stimulus in each condition was morphed into an average “old” face. We refer to these morphed conditions as “old on” (Fig. 1b, lower row). The amount by which each stimulus was morphed into the average “old” face was set to 0.35, ensuring that the “old off” faces were highly distinct from the “old on” ones (see Supplementary Information). These “old” faces were shown randomly intermixed within all blocks. Note that for the “old on” faces, only the form but not the motion was morphed into the average “old” face. As the presence of an “old” morph did not affect the reliability of the motion information (*z* = −0.60, *p* > 0.250, two-sided Wilcoxon signed-ranked test on fitted standard deviations; see Supplementary Information), we collapsed “old on” and “old off” in the motion condition for later analysis.

### Models

We used three models that differ in how facial form and motion are integrated based on subjects’ identity choices. Each model consists of an encoding stage (generative model) and a decision stage. In the decision stage, the observer applies a decision rule to determine their response, “Laura” or “Susan”. The models that we tested only differ in that decision rule.

### Encoding stage (generative model)

The generative model describes the task statistics and the observer’s measurement noise.

We focus on two high-level visual cues, facial motion and form, simplifying them as one-dimensional, i.e., projections onto a one-dimensional axis connecting Laura and Susan. On both these axes, we represent Laura by 0 and Susan by 1. Each trial is then characterized by a motion morph parameter *s*_m_ and a form morph parameter *s*_f_ (both between 0 and 1) (Fig. 1a). Furthermore, each trial is characterized by the occurrence of “old” (i.e., old on/off) denoted by a categorical variable *c* taking values 0 and 0.35 (Fig. 1b). As described above, the value of 0.35 was chosen based on preliminary testing during the familiarization phase so that subjects clearly perceived the faces as “old” but were still able to discriminate Laura from Susan. In “old on” conditions, form was a mix consisting of 0.65 of *s*_f_ and 0.35 of *s*_f_ of the “old” perceptual average. Since the average “old” face consisted of 0.4 Laura and 0.6 Susan, as described above, the *s*_f_ of the old average face was 0.6. Generally, we denote the form stimulus is 0.6*c* + (1 − *c*)*s*_f_, where *c* = 0 in “old off”, and *c* = 0.35 in “old on”. During the experiment, three stimulus types are known to the subject: (1) motion-only, where *s*_f_ = 0.5, (2) form-only, where *s*_m_ = 0.5, and (3) combined. However, the subject did not know that the combined-cue condition was subdivided into congruent trials, when *s*_m_ = *s*_f_, and incongruent trials, when *s*_m_ and *s*_f_ differed by an amount Δ of 0.15, with either *s*_m_ = *s*_f_ +Δ (which we call the +Δ condition) or *s*_m_ = *s*_f_ − Δ (the −Δ condition).

At least two sources of noise could play a role: measurement noise, and memory noise associated with the prototypes of Laura and Susan. For simplicity, we assume that although the prototype memories will be noisy, the specific memories will not change much over trials, and thus its predominant effect is in bias, not variance. We denote the noisy measurements of each feature by *x*_m_ and *x*_f_ for motion and form, respectively. We assume that these measurements are conditionally independent given *s*_m_ and *s*_f_, and follow Gaussian distributions:

$$p({x}_{f}|{s}_{f})=\frac{1}{\sqrt{2{\rm{\pi }}{\sigma }_{{\rm{f}}}^{2}}}{e}^{-\frac{{({x}_{{\rm{f}}}-[0.6c+(1-c){s}_{{\rm{f}}}])}^{{\rm{2}}}}{2{\sigma }_{{\rm{f}}}^{2}}}.$$

We allow the noise level for form, *σ*_f_, to be different in “old on” and “old off” conditions. This could reflect the possibility that establishing the identity of the “old” faces requires an extrapolation process that introduces extra variability.

### Decision stage

Next, we model the observer’s inference process. The optimal model is largely identical to the optimal model in earlier cue combination studies^6, 7^. The optimal observer computes the probability of a stimulus *s* given the noisy measurements *x*_m_ and *x*_f_. We make the common assumption that the observer acts as if they believe that there is only a single *s* to be inferred; this is somewhat plausible since no subject reported noticing a conflict.

We denote the likelihood ratio over face category as follows:

$$\frac{L({\rm{Susan}})}{L({\rm{Laura}})}=\frac{{\int }_{b}^{\infty }p({x}_{{\rm{m}}},{x}_{{\rm{f}}}|s)ds}{{\int }_{-\infty }^{b}p({x}_{{\rm{m}}},{x}_{{\rm{f}}}|s)ds},$$

where *p*(*x*_m_ *, x*_f_|s) is the probability of *s* under the noisy measurements *x*_m_ and *x*_f_, and *b* the category boundary. The integrand of both the numerator and the denominator as the likelihood function over the underlying continuous morph level *s* (rather than over identity, which is categorical), and we denote it by *L*_*s*_(*s*)≡*p*(*x*_m_,*x*_f_|*s*).

The optimal (accuracy-maximizing) observer would report “Susan” when *L*(Susan) > *L*(Laura). This happens if and only if the median of the likelihood function *L*_*s*_(*s*) excee﻿ds *b*. We now introduce the notation *N*(*y*; *μ*, *σ* ^2^) for a normal distribution over *y* with mean *μ* and variance *σ* ^2^. If the observer knows what *c* is in any given trial, then the likelihood function o﻿ver﻿ *s* ﻿can be evaluated as

$${L}_{s}(s)\propto {\rm{N}}(s;\frac{{J}_{{\rm{m}}}{x}_{{\rm{m}}}+(1-c){J}_{{\rm{f}}}({x}_{{\rm{f}}}-0.6c)}{{J}_{{\rm{m}}}+{(1-c)}^{2}{J}_{{\rm{f}}}},\frac{1}{{J}_{{\rm{m}}}+{(1-c)}^{2}{J}_{{\rm{f}}}}),$$

where we introduced notation for precision: ${J}_{{\rm{m}}}\equiv \frac{1}{{\sigma }_{{\rm{m}}}^{2}}$ and ${J}_{{\rm{f}}}\equiv \frac{1}{{\sigma }_{{\rm{f}}}^{2}}$. In the special case that *c* = 0, *L*_*s*_(*s*) reduces to the common expression for integrated likelihoods^1^.

Since the median of a normal distribution is the same as its mean, the optimal decision rule for an observer is to report “Susan” when

1 $$\frac{{J}_{{\rm{m}}}{x}_{{\rm{m}}}+\,(1-c){J}_{{\rm{f}}}({x}_{{\rm{f}}}-0.6c)}{{J}_{{\rm{m}}}+\,{(1-c)}^{2}{J}_{{\rm{f}}}} > b{\rm{.}}$$

In the best-cue model, the observer only relies on the cue with the highest *J*. Thus, the decision rule for when to report “Susan”, equation (1), gets replaced by

2 $$\begin{array}{c}{x}_{{\rm{m}}} > b\,{\rm{if}}\,{\sigma }_{{\rm{m}}} < {\sigma }_{{\rm{f}}}\\ {x}_{{\rm{f}}} > b\,{\rm{if}}\,{\sigma }_{{\rm{m}}} > {\sigma }_{{\rm{f}}}{\rm{.}}\end{array}$$

In the simple-average model, the observer responds “Susan” when

3 $$\frac{{x}_{{\rm{m}}}+\,{x}_{{\rm{f}}}}{2} > b{\rm{.}}$$

### Experimental predictions

Finally, we derive experimental predictions for each of the models, based on their respective decision rules (equations (1), (2), (3)). To this end, we need the probability that the decision rule returns “Susan” for a given experimental condition (which is characterized by *s*_m_, *s*_f_, and *c*).

In the optimal model, the probability of responding “Susan” is:

$$Pr({\rm{r}}{\rm{e}}{\rm{s}}{\rm{p}}{\rm{o}}{\rm{n}}{\rm{d}}\,{\textstyle \text{``}}{\rm{S}}{\rm{u}}{\rm{s}}{\rm{a}}{\rm{n}}{\textstyle \text{''}}|{s}_{{\rm{m}}},{s}_{{\rm{f}}},c,\lambda )=\frac{\lambda }{2}+(1-\lambda ){\rm{\Phi }}(\frac{{J}_{{\rm{m}}}({s}_{{\rm{m}}}-b)+\,{(1-c)}^{2}{J}_{{\rm{f}}}({s}_{{\rm{f}}}-b)}{\sqrt{{J}_{{\rm{m}}}+\,{(1-c)}^{2}{J}_{{\rm{f}}}}})\,,$$

where Φ is the conventional notation for the cumulative standard normal distribution (in Matlab: normcdf(…, 0, 1)), and *λ* the probability that the subject guesses randomly.

In the best-cue model, the left-hand side of equation (1) has mean *s*_m_ and variance *σ*_m_^2^ if *σ*_m_ < *σ*_f_, and mean 0.6*c* + (1 − *c*)*s*_f_ and variance *σ*_f_^2^ if *σ*_m_ > *σ*_f_. The response probabilities are given by

$$Pr({\rm{r}}{\rm{e}}{\rm{s}}{\rm{p}}{\rm{o}}{\rm{n}}{\rm{d}}\,\,{\textstyle \text{``}}{\rm{S}}{\rm{u}}{\rm{s}}{\rm{a}}{\rm{n}}{\textstyle \text{''}}{s}_{{\rm{m}}},{s}_{{\rm{f}}},c,\lambda )=\{\begin{array}{c}\frac{\lambda }{2}+(1-\lambda ){\rm{\Phi }}(\frac{{s}_{{\rm{m}}}-b}{{\sigma }_{{\rm{m}}}})\,{\rm{i}}{\rm{f}}\,{\sigma }_{{\rm{m}}} < {\sigma }_{{\rm{f}}}\\ \frac{\lambda }{2}+(1-\lambda ){\rm{\Phi }}(\frac{0.6c+(1-c){s}_{{\rm{f}}}-b}{{\sigma }_{{\rm{f}}}})\,{\rm{i}}{\rm{f}}\,{\sigma }_{{\rm{m}}} > {\sigma }_{{\rm{f}}}\end{array}$$

In the simple-average model, the probability of responding “Susan” is

$$Pr({\rm{r}}{\rm{e}}{\rm{s}}{\rm{p}}{\rm{o}}{\rm{n}}{\rm{d}}\,\,{\textstyle \text{``}}{\rm{S}}{\rm{u}}{\rm{s}}{\rm{a}}{\rm{n}}{\textstyle \text{''}}| {s}_{{\rm{m}}},{s}_{{\rm{f}}},c,\lambda )=\frac{\lambda }{2}+(1-\lambda ){\rm{\Phi }}(\frac{{s}_{{\rm{m}}}+0.6c+(1-c){s}_{{\rm{f}}}-2b}{\sqrt{{{\sigma }_{{\rm{m}}}}^{2}+{{\sigma }_{{\rm{f}}}}^{2}}})$$

### Model fitting and model comparison

We fitted all parameters using maximum-likelihood estimation for each model and each subject. Parameter fitting was implemented using the Matlab function *fmincon* with 10 different random initializations per optimization. To verify that our fitting procedure could recover the model parameters, we performed parameter recovery. To validate our model comparison process, we performed model recovery on synthetic data sets. In addition to the joint fitting based on all data, we performed a predictive analysis using only the data from the single-cue conditions to fit all parameters and tested those on the combined conditions. We used non-parametric tests on the computed maximum log likelihoods to test for differences between models. Moreover, we used a random-effects method for Bayesian model selection at the group level^15^.

Further details and derivations, as well as equations for the optimal model with incorrect beliefs are provided in the Supplementary Information.

## Electronic supplementary material

Supplementary Information
