# Supplementary material for: Near-optimal integration of facial form and motion
Source: Sci Rep. 2017 Sep 8;7:11002. doi: 10.1038/s41598-017-10885-y (PMC5591281; doi:10.1038/s41598-017-10885-y)
Supplement: Supplementary file 1 — Supplementary Information [file 41598_2017_10885_MOESM1_ESM.pdf]

Near-optimal integration of facial form and motion

Katharina Dobs[1,2*], Wei Ji Ma[3] and Leila Reddy[1,2]

[1] Université de Toulouse, Centre de Recherche Cerveau et Cognition, Université Paul Sabatier, Toulouse, France.

[2] CNRS, UMR 5549, Faculté de Médecine de Purpan, Toulouse, France.

[3] New York University, Center for Neural Science and Department of Psychology, New York, New York, USA.

SUPPLEMENTARY METHODS AND RESULTS

EXPERIMENTAL METHODS AND RESULTS

Design and procedure

The experimental procedure consisted of three phases: familiarization, training, and testing.

Familiarization phase

Procedure. During the familiarization phase, which lasted about 10 minutes, subjects performed a same-different task on either Laura or Susan to familiarize themselves with the basic facial forms and their facial movements. Subjects performed two blocks of 60 trials, one for each facial identity, in randomized order. Each trial began with 0.5 s fixation period in which a fixation cross was centrally presented, followed by two 1 s dynamic face stimuli with a 0.1 s fixation period in between stimuli to avoid effects of apparent motion. The first stimulus always showed one basic facial identity (e.g., Laura's facial form and motion; Fig. 1B "old off"), followed by a second stimulus that was either the same or morphed by a certain amount (i.e., 0.05 morph level steps between 0.05 and 1) into the "old" face (e.g., Laura's "old" facial form and motion; Fig. 1B "old on"). The amount of morph level was controlled by a Quest staircase procedure (initial threshold: 0.35), which ensured that subjects maintained a performance of 70.7% correct on "different" trials. One third of the trials were "same" trials. After the second stimulus, subjects had a maximum of 3 s to respond "same" or "different" by a button press (left and right arrow, respectively). At the end of each trial, subjects received feedback ("correct", "wrong", or "too late") shown for 0.3 s on the screen.

Results. The estimated threshold (i.e., morph level) to detect a change was 0.30 (IQR: [0.23, 0.37]) for Laura and 0.30 (IQR: [0.26, 0.42]) for Susan. The thresholds did not differ between identities ($z = -0.76$, $p > .250$, two-sided Wilcoxon signed-ranked test).

Training phase

Procedure. In the training phase, which also lasted about 10 minutes, subjects performed an identity discrimination task on the two previously learned identities based on form, motion or both cues combined, tested in three separate blocks. Each

block contained 40 trials, half of which showed Laura and half Susan. The order of form and motion blocks was randomized across subjects, and the combined block was always shown last. At the beginning of each block, subjects were informed about the type of the block. In form blocks, subjects were asked to discriminate the face stimuli solely based on facial form and they were informed that facial motion is uninformative (i.e., the average between both facial motions), and vice versa for motion blocks. During combined blocks, subjects had to discriminate the face stimuli based on facial form and motion. At the beginning of each trial, a short cue (letter "F", "M", or "C" for form, motion and combined blocks, respectively) was presented for 0.3 s to remind subjects of the block type (i.e., the task to perform), followed by a 0.2 s fixation cross centred on the screen. Following the fixation period, a face stimulus was shown for 1 s showing either Laura or Susan with their basic facial form (100% facial form, average facial motion), facial motion (100% facial motion, average facial form) or both (100% facial form and motion). A response screen ("Laura or Susan?") appeared after the stimulus either until a response was recorded (left or right arrow for Laura or Susan, respectively) or until a maximum duration of 2 s was reached. Subjects could respond during the stimuli presentation (in which case the response screen did not appear) or during the presentation of the response screen. At the end of each trial, feedback ("correct", "wrong", or "too late") was shown for 0.5 s on the screen. Note that in the training phase, we only showed the basic face stimuli (i.e., 100%) and subjects were never shown any of the intermediate morph stimuli or the "old" morphs.

Results. Subjects could perfectly discriminate the two facial identities based on facial form (0.99, [0.98, 1] (median proportion correct, IQR across subjects)), facial motion (0.96, [0.93, 0.98]) and both cues (1.0, [1.0, 1.0]).

Testing phase

During the testing phase, which lasted about 70 minutes, subjects had to categorize face stimuli to Laura or Susan based on form, motion or both cues combined in separate blocks (Fig. 1C), similar to the training phase. Subjects performed five form blocks, five motion blocks and 14 combined blocks in randomized order, and were informed about the type at the beginning of each block. In contrast to the training phase, intermediate morph levels and "old" morphs were shown in addition to the basic face stimuli. Subjects were explicitly told about the

occurrence of intermediate and “old” morphs. The trial sequence was the same as for the training phase, except that no feedback was provided at the end of a trial. In form and motion blocks (Fig. 1A, “Form”, “Motion”), the basic face stimuli (morph levels 0 and 1), the nine intermediate morph levels (0.2, 0.3, 0.4, 0.45, 0.5, 0.55, 0.6, 0.7, 0.8), and the “old” morphs at all 11 morph levels were each presented three times for a total of 66 trials per block (11 morph levels x 2 old on/off x 3 repetitions).

Combined blocks contained 22 “congruent” (Fig. 1A, “Comb”) and 44 “incongruent” trials (Fig. 1A, “Comb, +Δ”, “Comb, −Δ”). On “congruent” trials, a common morph level was chosen for form and motion from one of the 11 values listed above. Each face stimulus was presented only once, for a total of 22 “congruent” trials per block (11 morph levels x 2 old on/off). On “incongruent” trials, we showed face stimuli that had different morph levels for form and motion: when the original morph level was s, the form morph level was $s+\Delta/2$ and the motion morph level was $s-\Delta/2$. In “Comb, +Δ” trials, Δ was 0.15, and in “Comb, −Δ” trials, Δ was −0.15. To allow for such incongruence also at the lowest and highest morph levels, we replaced, only in the “incongruent” trials, the 0 and 1 morph levels by 0.1 and 0.9, respectively. Each face stimulus was presented once, yielding 44 “incongruent” trials (11 morph levels x 2 old on/off x 2 values of Δ) per block. Note that subjects were not aware of the presence of “incongruent” trials.

DERIVATIONS OF MODEL PREDICTIONS

General model structure

Each model consists of an encoding stage (generative model) and a decision stage. In the decision stage, the observer applies a decision rule to determine their response, “Laura” or “Susan”. The models that we tested only differ in that decision rule. One of the models we test uses an optimal decision rule. Although this model is similar to what has been widely used [1,2], the underlying assumptions are worth spelling out, especially because we use a binary categorization task, and because the derivation needs to be modified for the suboptimal decision rules.

Encoding stage (generative model)

The generative model describes the task statistics and the observer’s measurement noise.

Task statistics. Each trial is characterized by a motion morph parameter s_{m} and a form morph parameter s_{f} (both between 0 and 1) (Fig. 1A). Furthermore, each trial is characterized by the occurrence of "old" (i.e., old on/off) denoted by a categorical variable c taking values 0 and 0.35 (Fig. 1B). As described above, the value of 0.35 was chosen based on preliminary testing during the familiarization phase so that subjects clearly perceived the faces as "old" but were still able to discriminate Laura from Susan. In "old on" conditions, form was a mix consisting of 0.65 of s_{f} and 0.35 of s_{f} of the "old" perceptual average. Since the average "old" face consisted of 0.4 Laura and 0.6 Susan, the s_{f} of the old average face was 0.6. Generally, we denote the form stimulus is $0.6c+(1-c)s_{\mathrm{f}}$, where $c=0$ in "old off", and $c=0.35$ in "old on". During the experiment, three stimulus types are known to the subject: (1) motion-only, where $s_{\mathrm{f}}=0.5$, (2) form-only, where $s_{\mathrm{m}}=0.5$, and (3) combined. However, the subject did not know that the combined-cue condition was subdivided into congruent trials, when $s_{\mathrm{m}}=s_{\mathrm{f}}$, and incongruent trials, when s_{m} and s_{f} differed by an amount Δ of 0.15, with either $s_{\mathrm{m}}=s_{\mathrm{f}}+\Delta$ (which we call the $+\Delta$ condition) or $s_{\mathrm{m}}= s_{\mathrm{f}}-\Delta$ (the $-\Delta$ condition).

Measurement noise. We denote the noisy measurements of each feature by x_{m} and x_{f} for motion and form, respectively. We assume that these measurements are conditionally independent given s_{m} and s_{f}, and follow Gaussian distributions:

$$
\begin{aligned}
p\left(x_{\mathrm{m}},x_{\mathrm{f}}\,|\,s_{\mathrm{m}},s_{\mathrm{f}}\right) &= p\left(x_{\mathrm{m}}\,|\,s_{\mathrm{m}}\right)p\left(x_{\mathrm{f}}\,|\,s_{\mathrm{f}}\right),\\
p\left(x_{\mathrm{m}}\,|\,s_{\mathrm{m}}\right) &= \frac{1}{\sqrt{2\pi\sigma_{\mathrm{m}}^{2}}}e^{-\frac{\left(x_{\mathrm{m}}-s_{\mathrm{m}}\right)^{2}}{2\sigma_{\mathrm{m}}^{2}}},\\
p\left(x_{f}\,|\,s_{f}\right) &= \frac{1}{\sqrt{2\pi\sigma_{\mathrm{f}}^{2}}}e^{-\frac{\left(x_{\mathrm{f}}-\left[0.6c+(1-c)s_{\mathrm{f}}\right]\right)^{2}}{2\sigma_{\mathrm{f}}^{2}}}.
\end{aligned} \tag{1}
$$

Decision stage

Optimal model. Next, we model the observer's inference process. The optimal model is largely identical to the optimal model in earlier cue combination studies [1,2] . However, it is worth spelling out the assumptions; moreover, some details are specific to our design. The optimal observer computes the probability of a stimulus s given the noisy measurements x_{m} and x_{f}. We make the common assumption that the observer acts as if they believe that there is only a single s to be inferred; this is somewhat plausible since no subject reported noticing a conflict.

We denote the likelihood ratio over face category as follows:

$$\frac{L(\text{Susan})}{L(\text{Laura})}=\frac{p(x_{\text{m}},x_{\text{f}}|\text{Susan})}{p(x_{\text{m}},x_{\text{f}}|\text{Laura})}=\frac{\int p(x_{\text{m}},x_{\text{f}}|s)\,p(s|\text{Susan})\,ds}{\int p(x_{\text{m}},x_{\text{f}}|s)\,p(s|\text{Laura})\,ds}, \tag{2}$$

where $p(s|\text{Susan})$ or $p(s|\text{Laura})$ is the probability of s under Susan or Laura, respectively. We assume that the observer believes these distributions of s to be uniform on a large interval from $-a$ to some category boundary b (Laura) and from the same b to a (Susan). Then,

$$p(s|\text{Laura})=\begin{cases}\dfrac{1}{a+b} & \text{if s} < b\\ 0 & \text{otherwise}\end{cases}$$

$$p(s|Susan)=\begin{cases}\dfrac{1}{a-b} & \text{if } s > b\\ 0 & \text{otherwise}\end{cases}$$

We assume $a \gg b$ so that we can make the approximation

$$p(s|\text{Laura})\approx\begin{cases}\dfrac{1}{a} & \text{if s} < b\\ 0 & \text{otherwise}\end{cases}$$

$$p(s|Susan)\approx\begin{cases}\dfrac{1}{a} & \text{if } s > b\\ 0 & \text{otherwise}\end{cases}$$

Then, equation (2) simplifies to

$$\frac{L(\text{Susan})}{L(\text{Laura})}=\frac{\int_b^\infty p(x_{\text{m}},x_{\text{f}}|s)\,ds}{\int_{-\infty}^b p(x_{\text{m}},x_{\text{f}}|s)\,ds}, \tag{3}$$

The optimal (accuracy-maximizing) observer would report "Susan" when $L(\text{Susan})>L(\text{Laura})$. According to equation (3), this is equivalent to

$$\int_b^\infty p(x_{\text{m},}x_{\text{f}}|s)\,ds > \int_{-\infty}^b p(x_{\text{m},}x_{\text{f}}|s)\,ds,$$

or in other words, when the median of the (normalized) likelihood function over s, which we define as $L_s(s)=p(x_{\text{m}},x_{\text{f}}|s)$, exceeds b. We now introduce the notation $N(y;\mu,\sigma^2)$ for a normal distribution over y with mean μ and variance σ^2. We assume that the observer knows c is on any trial. Then, the likelihood function $L_s(s)$ can be evaluated as

$$
\begin{aligned}
L_s &= p\left(x_{\mathrm{m}}, x_{\mathrm{f}} \middle| s\right) \\
&= p\left(x_{\mathrm{m}} \middle| s\right) p\left(x_{\mathrm{f}} \middle| s\right) \\
&= \mathrm{N}\left(x_{\mathrm{m}}; s, \sigma_{\mathrm{m}}^2\right) \mathrm{N}\left(x_{\mathrm{f}}; 0.6c + (1-c)s, \sigma_{\mathrm{f}}^2\right) \\
&= \mathrm{N}\left(s; x_{\mathrm{m}}, \sigma_{\mathrm{m}}^2\right) \mathrm{N}\left(0.6c + (1-c)s; x_{\mathrm{f}}, \sigma_{\mathrm{f}}^2\right) \\
&= \mathrm{N}\left(s; x_{\mathrm{m}}, \sigma_{\mathrm{m}}^2\right) \mathrm{N}\left((1-c)s; x_{\mathrm{f}} - 0.6c, \sigma_{\mathrm{f}}^2\right) \\
&\propto \mathrm{N}\left(s; x_{\mathrm{m}}, \sigma_{\mathrm{m}}^2\right) \mathrm{N}\left(s; \frac{x_{\mathrm{f}} - 0.6c}{1-c}, \frac{\sigma_{\mathrm{f}}^2}{(1-c)^2}\right) \\
&\propto \mathrm{N}\left(s; \frac{\frac{x_{\mathrm{m}}}{\sigma_{\mathrm{m}}^2} + \frac{(1-c)(x_{\mathrm{f}} - 0.6c)}{\sigma_{\mathrm{f}}^2}}{\frac{1}{\sigma_{\mathrm{m}}^2} + \frac{(1-c)^2}{\sigma_{\mathrm{f}}^2}}, \frac{1}{\frac{1}{\sigma_{\mathrm{m}}^2} + \frac{(1-c)^2}{\sigma_{\mathrm{f}}^2}}\right) \\
&= \mathrm{N}\left(s; \frac{J_{\mathrm{m}} x_{\mathrm{m}} + (1-c) J_{\mathrm{f}} (x_{\mathrm{f}} - 0.6c)}{J_{\mathrm{m}} + (1-c)^2 J_{\mathrm{f}}}, \frac{1}{J_{\mathrm{m}} + (1-c)^2 J_{\mathrm{f}}}\right),
\end{aligned} \tag{4}
$$

where we used the assumption of conditional independence in going from line 1 to line 2, absorbed s-independent factors into the proportionality sign in the second-to-last line, and introduced notation for precision: $J_{\mathrm{m}} \equiv \frac{1}{\sigma_{\mathrm{m}}^2}$ and $J_{\mathrm{f}} \equiv \frac{1}{\sigma_{\mathrm{f}}^2}$. In the special case that c=0, the likelihood $L_s(s)$ reduces to the common expression for integrated likelihoods [3].

Since the median of a normal distribution is the same as its mean, the optimal decision rule for an observer is to report "Susan" when

$$
\frac{J_{\mathrm{m}} x_{\mathrm{m}} + (1-c) J_{\mathrm{f}} (x_{\mathrm{f}} - 0.6c)}{J_{\mathrm{m}} + (1-c)^2 J_{\mathrm{f}}} > b \ . \tag{5}
$$

Optimal model with incorrect beliefs. We now consider a variant of the optimal model. The optimal observer possesses and utilizes complete knowledge of the task structure. However, at least one aspect of this knowledge is rather unrealistic, namely the knowledge that the "old" face is a morph between Laura and Susan. Human observers might therefore behave as if they do not have this knowledge and instead assume that the "old" version of Laura is pure Laura (instead of being morphed into the "old" average of Laura and Susan), and the "old" version of Susan is pure Susan. Then, the *assumed* noise distributions will be

$$p_{\text{assumed}}\left(x_{\text{m}}|s\right)=\frac{1}{\sqrt{2\pi\sigma_{\text{m}}^{2}}}e^{-\frac{\left(x_{\text{m}}-s\right)^{2}}{2\sigma_{\text{m}}^{2}}}$$

$$p_{\text{assumed}}\left(x_{\text{f}}|s\right)=\frac{1}{\sqrt{2\pi\sigma_{\text{f}}^{2}}}e^{-\frac{\left(x_{\text{f}}-s\right)^{2}}{2\sigma_{\text{f}}^{2}}}$$

which corresponds to assuming that c=0 even though in reality it is not.
As a consequence, the decision rule, equation (5) simplifies to

$$\frac{J_{\text{m}}x_{\text{m}}+J_{\text{f}}x_{\text{f}}}{J_{\text{m}}+J_{\text{f}}}>b \tag{6}$$

Best-cue model. In the best-cue model, the observer only relies on the cue with the highest J. Thus, the decision rule Eq. (5) gets replaced by

$$\begin{aligned} x_{\text{m}} &> b \quad \text{if } \sigma_{\text{m}}<\sigma_{\text{f}} \\ x_{\text{f}} &> b \quad \text{if } \sigma_{\text{m}}>\sigma_{\text{f}} \end{aligned} \tag{7}$$

Simple-average model. In the simple-average model, the observer responds "Susan" when

$$\frac{x_{\text{m}}+x_{\text{f}}}{2}>b\ . \tag{8}$$

Experimental predictions

Finally, we derive experimental predictions for each of the models, based on their respective decision rules. To this end, we need the probability that the decision rule returns "Susan" for a given experimental condition (which is characterized by s_{m}, s_{f}, and c). These probabilities are obtained by integrating over x_{m} and x_{f} under their distributions $p(x_{\text{m}}|s_{\text{m}})$ and $p(x_{\text{f}}|s_{\text{f}})$.

Optimal model. In the optimal model, the left-hand side of the decision rule is normally distributed with mean $\frac{J_{\text{m}}s_{\text{m}}+\left(1-c\right)^{2}J_{\text{f}}s_{\text{f}}}{J_{\text{m}}+\left(1-c\right)^{2}J_{\text{f}}}$, and standard deviation $\frac{1}{\sqrt{J_{\text{m}}+\left(1-c\right)^{2}J_{\text{f}}}}$. Therefore, the probability of responding "Susan" is:

$$\Pr\left(\text{respond "Susan"}|s_{\text{m}},s_{\text{f}},c\right)=\Phi\left(\frac{J_{\text{m}}s_{\text{m}}+\left(1-c\right)^{2}J_{\text{f}}s_{\text{f}}-b}{\sqrt{J_{\text{m}}+\left(1-c\right)^{2}J_{\text{f}}}}\right)\ ,$$

where Φ is the conventional notation for the cumulative standard normal distribution (in Matlab: normcdf(…,0,1)). Finally, if the subject guesses randomly with probability λ, the probability of responding "Susan" becomes

$$\Pr\left(\text{respond "Susan"}\middle|s_{\text{m}},s_{\text{f}},c,\lambda\right)=\frac{\lambda}{2}+\left(1-\lambda\right)\Phi\left(\frac{J_{\text{m}}\left(s_{\text{m}}-b\right)+\left(1-c\right)^{2}J_{\text{f}}\left(s_{\text{f}}-b\right)}{\sqrt{J_{\text{m}}+\left(1-c\right)^{2}J_{\text{f}}}}\right) \quad (9)$$

Optimal model with incorrect beliefs. We follow the same logic as in the optimal model, but now with a different decision rule, equation (6). The left-hand side of that equation has mean $\frac{J_{\text{m}}s_{\text{m}}+J_{\text{f}}\left(0.6c+\left(1-c\right)s_{\text{f}}\right)}{J_{\text{m}}+J_{\text{f}}}$ and variance $\frac{1}{J_{\text{m}}+J_{\text{f}}}$. Thus, we find for the probability of responding "Susan",

$$\Pr\left(\text{respond "Susan"}\middle|s_{\text{m}},s_{\text{f}},c,\lambda\right)=\frac{\lambda}{2}+\left(1-\lambda\right)\Phi\left(\frac{\frac{J_{\text{m}}s_{\text{m}}+J_{\text{f}}\left(0.6c+\left(1-c\right)s_{\text{f}}\right)}{J_{\text{m}}+J_{\text{f}}}-b}{\sqrt{\frac{1}{J_{\text{m}}+J_{\text{f}}}}}\right) \quad (10)$$

Note that we have used the distributions of x_{m} and x_{f} from the generative model, equations (1) to derive this expression.

Best-cue model. The left-hand side of Eq. (7) has mean s_{m} and variance σ_{m}^2 if $\sigma_{\text{m}}<\sigma_{\text{f}}$, and mean $0.6c + (1-c)s_{\text{f}}$ and variance σ_{f}^2 if $\sigma_{\text{m}}>\sigma_{\text{f}}$. The response probabilities are given by

$$\Pr\left(\text{respond "Susan"}\middle|s_{\text{m}},s_{\text{f}},c,\lambda\right)=\begin{cases}\frac{\lambda}{2}+\left(1-\lambda\right)\Phi\left(\frac{s_{\text{m}}-b}{\sigma_{\text{m}}}\right) & \text{if } \sigma_{\text{m}}<\sigma_{\text{f}}\\ \frac{\lambda}{2}+\left(1-\lambda\right)\Phi\left(\frac{0.6c+\left(1-c\right)s_{\text{f}}-b}{\sigma_{\text{f}}}\right) & \text{if } \sigma_{\text{m}}>\sigma_{\text{f}}\end{cases} \quad (11)$$

Simple-average model. The left-hand side of Eq. (8) has mean $\frac{s_{\text{m}}+0.6c+\left(1-c\right)s_{\text{f}}}{2}$ and variance $\frac{\sigma_m^2+\sigma_f^2}{4}$, and therefore the probability of responding "Susan" is

$$\Pr\left(\text{respond "Susan"}\middle|s_{\text{m}},s_{\text{f}},c,\lambda\right)=\frac{\lambda}{2}+\left(1-\lambda\right)\Phi\left(\frac{s_{\text{m}}+0.6c+\left(1-c\right)s_{\text{f}}-2b}{\sqrt{\sigma_{\text{m}}^2+\sigma_{\text{f}}^2}}\right). \quad (12)$$

MODEL FITTING METHODS AND SUPPLEMENTARY RESULTS

Methods

In any model, the probability of responding "Susan" within a given trial depends on the form morph parameters s_{f} (or $s_{\mathrm{f,old}}$ in case of "old on"), the motion morph parameter s_{m}, and the model parameters. We denote the set of model parameters by θ. All models have the same set of parameters: the standard deviations σ_{m}, σ_{f} ($\sigma_{\mathrm{f,old}}$ in case of old off), the category boundary b, and a lapse rate λ (i.e., accounting for attentional fluctuations, eye blinks, etc.). The log likelihood of a specific combination of parameter values (not to be confused with the likelihoods in the observer model) is the probability of the observer's empirical responses given that parameter combination and is obtained as

$$\begin{aligned}\log L_{\mathrm{model}}(\theta) &= \log p(\mathrm{data}|\mathrm{stimuli},\theta) \\ &= \log \prod_{i=1}^{n_{\mathrm{trials}}} p(\mathrm{response}|\mathrm{s}_{\mathrm{f},i},\mathrm{s}_{\mathrm{m},i},\theta) \\ &= \log \sum_{i=1}^{n_{\mathrm{trials}}} \log p(\mathrm{response}|\mathrm{s}_{\mathrm{f},i},\mathrm{s}_{\mathrm{m},i},\theta),\end{aligned}$$

where n_{trials} is the total number of trials, $s_{\mathrm{f},i}$ and $s_{\mathrm{m},i}$ are the form and motion morph parameters on the ith trial, and $\mathrm{response}_i$ is the subject's response on the ith trial. The probabilities $p(\mathrm{response}_i|\ s_{\mathrm{f},i},\ s_{\mathrm{m},i},\ \theta)$ are obtained from equation (9), (10), (11) or (12). The maximum-likelihood estimates of the parameters are the combination of parameters θ that maximize log L_{model}. We fitted the data in the single-cue and combined-cue conditions jointly.

For maximizing the log likelihood, we used the Matlab function *fmincon*. We constrained the σ parameters to the interval (0,10], the category boundary b to [-10,10], and the lapse rate λ to [0,1]. To minimize the risk that *fmincon* finds a global instead of a local maximum, we ran the function ten times using different initial parameters drawn from Gaussian distributions with mean and standard deviation as estimated from preliminary testing. The fit that returned the highest log likelihood then served to provide the maximum-likelihood estimates of the parameters.

Parameter recovery

To test how well our fitting procedure could recover the model parameters, we generated 100 synthetic data sets of the same size as a subject data set. To create a synthetic data set, we randomly drew the value of each parameter from a normal distribution using the median value and the interquartile range obtained from the joint fitting as mean and standard deviation. We then simulated trial-to-trial responses from the model's probabilities of responding "Susan" given those parameter values and the same stimuli as used in the experiment. Finally, we fitted the model used to generate the data. Given that the number of trials is finite, we expect the log likelihood of the estimated parameters to be slightly higher than the true parameters. All parameters were well recovered (see Fig. S1) and, as predicted, the log likelihoods of the estimated parameters were slightly higher than of the true parameters, for the optimal (1.82, [1.11, 3.08] (median difference, IQR across subjects); $z = -8.68$, $p < .001$, two-sided Wilcoxon signed-ranked test), the best-cue (1.80, [0.97, 2.49]; $z = -8.68$, $p < .001$) and the simple-average model (2.06, [1.11, 2.96]; $z = -8.68$, $p < .001$).

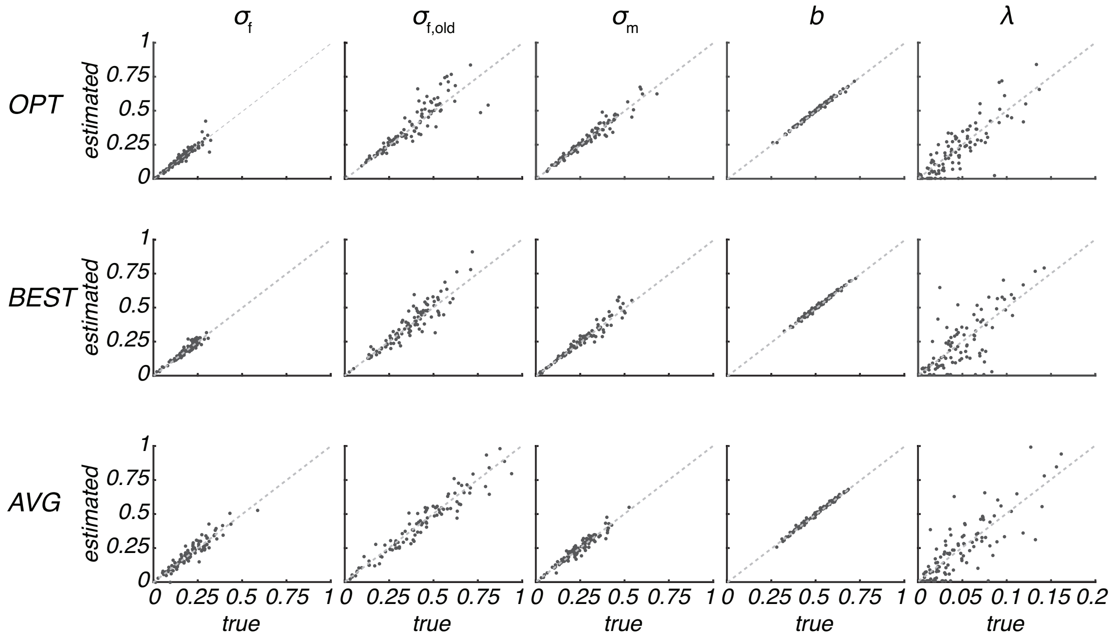

Fig. S1. *Parameter recovery.* The results of the parameter recovery for all five fitted parameters (σ_f, $\sigma_{f,old}$, σ_m, *b*, *λ;* columns from left to right) for the optimal (OPT; upper row), the best-cue (BEST; middle row) and the simple-average model (AVG; lower row). Each plot shows the estimated against the true parameter value in 100 synthetic data sets.

Single-cue fitting

We fitted all parameters based on the single-cue conditions using maximum-likelihood estimation. We assumed that the bias b and the lapse rate λ are shared across single-cue conditions. The standard deviations σ_{f} and $\sigma_{\mathrm{f,old}}$ were estimated from the form-only condition, σ_{m} from the motion-only condition. We implemented this fitting using nested *fmincon* functions in Matlab. We report the parameter estimates in Table S1.

Table S1. *Single-cue fitting.* Median and IQR across subjects (n = 22) of the maximum-likelihood parameter estimates obtained from the single-cue fitting.

**Parameter**	**Median**	**IQR**
σ_{f}	0.18	[0.14, 0.30]
$\sigma_{\mathrm{f,old}}$	0.21	[0.19, 0.27]
σ_{m}	0.23	[0.14, 0.29]
b	0.51	[0.45, 0.57]
λ	0.02	[0.00, 0.06]

The "old on" form manipulation reduced form reliability, as confirmed by a smaller estimated standard deviation for form in the "old off" than in the "old on" condition (-0.03, [-0.06, 0.01] (median difference, IQR)), although only marginally significant ($z = -1.33$, $p = .092$; one-sided Wilcoxon signed-rank test). To validate that the "old on" condition did not affect the motion discriminability, we further fitted $\sigma_{\mathrm{m},}$ b and λ in the motion-only condition separately for "old on" and "old off" faces. There was no significant difference between estimated standard deviations for "old on" and "old off" (0.01, [-0.09, 0.05]; $z = -0.60$, $p > .250$, two-sided Wilcoxon signed-ranked test). We thus collapsed "old on" and "old off" in the motion-only condition for later analyses.

MODEL COMPARISON METHODS AND SUPPLEMENTARY RESULTS

Methods

For each subject and each model, we calculated the maximum of the parameter log likelihood. We used non-parametric Wilcoxon signed-rank tests on

these maximum log likelihoods to test for differences between models. In addition, we used a random-effects method for Bayesian model selection at the group level [4].

Model recovery

To validate our model comparison process, we used the same synthetic data sets as for the parameter recovery but also fitted the models other than the one used to generate the data. For the data sets generated from the optimal model, the best-cue model fitted worse by 5.94, [1.58, 10.69] (median difference, IQR across subjects) of log likelihood, and the simple-average model by 10.43, [2.96, 20.05]. For the data sets generated from the best-cue model, the optimal model fitted worse by 5.68, [1.21, 10.37] of log likelihood, and the simple-average model by 19.37, [11.98, 31.55]. For the data sets generated from the simple-average model, the optimal model fitted worse by 5, [0, 13] of log likelihood, and the best-cue model by 14.70, [9.83, 24.14]. We further applied the random-effects method [4] to the log likelihoods obtained from fitting the synthetic data generated by one model to itself and the two other models. The model used to generate the synthetic data always reached a maximal protected exceedance probability of 1. This shows that our model comparison process recovers the correct model well if the true model is among the three models tested.

OPTIMAL MODEL WITH INCORRECT BELIEFS

We examined whether the optimal model with incorrect beliefs can better explain observers' behaviour than the optimal model. In particular, we fitted the parameters for this model using maximum likelihood estimation. The maximum-likelihood estimates of the parameters were 0.18, [0.15, 0.23] (median, IQR) for σ_{f}, 0.29, [0.21, 0.35] for $\sigma_{\mathrm{f,old}}$, 0.28, [0.21, 0.33] for σ_{m}, 0.51, [0.47, 0.56] for b, and 0.04, [0.01, 0.05] for λ. Figure S2 shows the fit of the optimal model with incorrect beliefs to the psychometric curves.

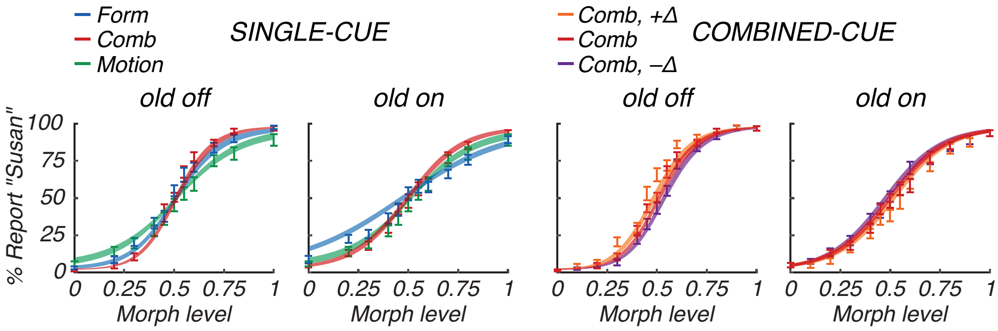

Fig. S2. *Optimal model with incorrect beliefs.* Mean percentage of "Susan" reports are shown for single cues ("Form" in blue, "Motion" in green; note that the combined-cue condition "Comb" in red is also shown for comparison) and for combined cues ("Comb" in red, "Comb, +Δ" in orange, and "Comb, −Δ" in purple), each separated for "old off" (first column) and "old on" (second column). Error bars and shaded areas represent ± 1 s.e.m. across subjects (n = 22), for data and model fit, respectively.

REACTION TIME ANALYSIS

Form and motion cues differ in how the available information develops over time (static form information is available from the beginning, while motion information evolves over time). To investigate how these inherent properties influence subjects' decision making in our task, subjects could freely choose when to make an identity choice, even during the presentation of the stimulus. Recent evidence has demonstrated that standard cue-integration models might be insufficient to explain cue integration behaviour in reaction-time tasks [5]. Thus, we examined reaction times in our experiment (Fig. S3). Visual inspection reveals that average reaction times depended on experimental condition, morph level and form reliability (i.e., old on/off). For all conditions, we can further see the typical inverse "U-shape" suggesting larger reaction times for intermediate morph levels than for morph levels at the outer bounds. To test for differences in reaction time for the experimental conditions, we performed multiple two-way (Condition x Morph level) repeated measures ANOVAs. In the single-cue conditions, we found a main effect of Morph level ("old off": $F(10,10) = 12.23$, $p < .001$, $\eta_p^2 = .21$; "old on": $F(10,10) = 6.49$, $p < .001$, $\eta_p^2 = .13$) supporting the inverse "U-shape" of reaction times. Furthermore, we found a significant effect of condition ("old off": $F(1,10) = 293.93$, $p < .001$, $\eta_p^2 =$

.39; “old on”: $F(1,10) = 128.03$, $p = .003$, $\eta_p^2 = .22$). During “old off”, the estimated values of the standard deviation parameter for facial form were larger than for facial motion (see “Single-cue fitting”) while reaction times were shorter, indicating a potential speed-accuracy trade-off.

Reaction times in the combined-congruent condition (“Comb” in red, left panels) significantly differed from those in the facial form condition in “old off” ($F(1,10) = 5.63$, $p = .018$, $\eta_p^2 = .01$) but not in “old on” ($F(1,10) = 2.14$, $p = .145$, $\eta_p^2 = .00$).

Next we analysed both the congruent and incongruent combined conditions. We did not consider the most extreme morph levels, as incongruent and congruent conditions differed at these morph levels (see Experimental Methods and Results above). As for single-cue conditions, we found a main effect of Morph level (“old off”: $F(8,8) = 36.23$, $p < .001$, $\eta_p^2 = .34$; “old on”: $F(8,8) = 10.28$, $p < .001$, $\eta_p^2 = .13$) indicating the inverse “U-shape” of reaction times. In contrast, reaction times did not differ between congruent and incongruent combined conditions (“Comb” in red, “Comb, +Δ” in orange, “Comb –Δ” in purple; right panels) for either “old off” ($F(2,8) = 0.56$, $p > .250$, $\eta_p^2 = .00$) or “old on” ($F(2,8) = 1.01$, $p > .250$, $\eta_p^2 = .00$). Thus we have no evidence that subjects treated these conditions differently, consistent with subjects’ reports during debriefing.

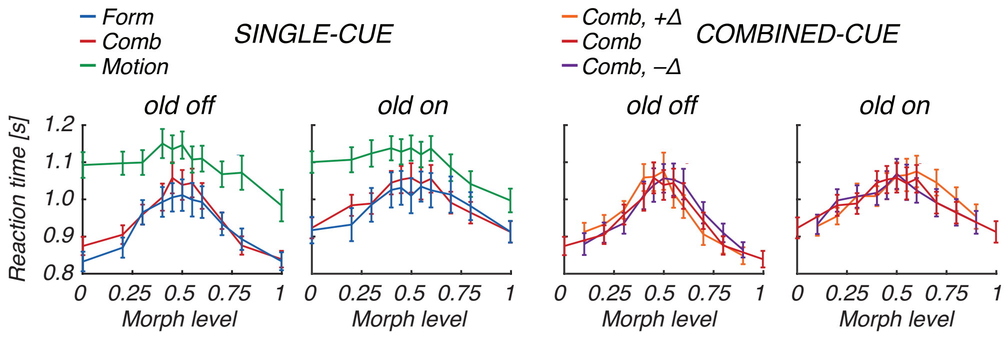

Fig. S3. *Reaction time analysis.* Mean reaction times are shown for single cues (“Form” in blue, “Motion” in green; note that the combined-cue condition “Comb” in red is also shown for comparison) and for combined cues (“Comb” in red, “Comb, +Δ” in orange, and “Comb, −Δ” in purple), each separated for “old off” (first column) and “old on” (second column). Error bars represent ± 1 s.e.m. across subjects (n = 22).

References

1. Ernst, M. O. & Banks, M. S. Humans integrate visual and haptic information in a statistically optimal fashion. *Nature* **415,** 429–433 (2002).
2. Alais, D. & Burr, D. The Ventriloquist Effect Results from Near-Optimal Bimodal Integration. *Curr. Biol.* **14,** 257–262 (2004).
3. Knill, D. C. & Richards, W. *Perception as Bayesian Inference*. (Cambridge University Press, 1996).
4. Rigoux, L., Stephan, K. E., Friston, K. J. & Daunizeau, J. Bayesian model selection for group studies - Revisited. *NeuroImage* **84,** 971–985 (2014).
5. Drugowitsch, J., DeAngelis, G. C., Klier, E. M., Angelaki, D. E. & Pouget, A. Optimal multisensory decision-making in a reaction-time task. *eLife* **3,** 1391–19 (2014).
